# Serotonin Signaling in Mouse Preimplantation Development: Insights from Transcriptomic and Structural-Functional Analyses

**DOI:** 10.3390/ijms252312954

**Published:** 2024-12-02

**Authors:** Veronika S. Frolova, Yulia O. Nikishina, Yuri B. Shmukler, Denis A. Nikishin

**Affiliations:** 1 Department of Embryology, Faculty of Biology, Lomonosov Moscow State University, Moscow 119234, Russia; frolova.veronika.2014@post.bio.msu.ru (V.S.F.); denisnikishin@gmail.com (D.A.N.); 2 Koltzov Institute of Developmental Biology, Russian Academy of Sciences, Moscow 119334, Russia; zubova.y@gmail.com

**Keywords:** serotonin signaling, vesicular monoamine transporter, serotonin receptors, oogenesis, preimplantation embryos, oocyte, blastocyst, intercellular signaling, reserpine, immunocytochemistry

## Abstract

Serotonin (5-HT), a versatile signaling molecule, plays a variety of roles in both neurotransmission and tissue regulation. The influence of serotonin on early development was first studied in marine invertebrate embryos and has since been documented in a variety of vertebrate species, including mammals. The present study investigates the expression and functional activity of serotonin components in mouse embryos, focusing on key receptors and transporters. Transcriptomic analyses revealed that mRNA transcripts related to serotonin show marked expression during the oogenesis and preimplantation stages. The results of the immunohistochemical studies show the presence of serotonin, the vesicular monoamine transporter VMAT2, and several membrane receptors (5-HT_1B_, 5-HT_1D_, 5-HT_2B_, 5-HT_7_) in the early stages of development. A functional analysis performed with the VMAT inhibitor reserpine revealed the crucial role of vesicular transport in the maintenance of serotonin signaling. The findings presented here support the hypothesis that serotonin plays a significant role in oocyte maturation and embryonic development, as well as in interblastomere interactions.

## 1. Introduction

Serotonin (5-HT) is a multifunctional signaling molecule, the role of which, as a neurotransmitter in the nervous system, represents only one particular manifestation of its diverse functions [1]. In the mammalian organism, a substantial proportion of this hormone is synthesized by enterochromaffin cells of the intestine, stored in platelets, and subsequently involved in a range of regulatory processes as a tissue hormone in the adult organism [2]. In the early 1960s, it was discovered that the functions of this signaling substance also come into play during embryonic development [3]. The serotonergic mechanisms are involved in such processes of early embryonic development as regulation of cell cycle dynamics, modulation of mechanical stiffness of the cytocortex, and realization of interblastomere interactions [4,5,6,7,8,9,10]. Subsequently, the presence and functional activity of serotonin, its transporters, and receptors have been demonstrated in the early stages of embryonic development in numerous vertebrate species, including mammals [9,11,12,13,14,15].

When studying this topic in the context of mammals, it is crucial that both oogenesis and embryogenesis take place within the maternal organism. Serotonin is transported from the maternal bloodstream to the ovary, where it accumulates in both the oocytes and the granulosa cells [16]. Serotonin has a positive effect on the activity of the follicle cells, which leads to increased concentrations of secondary messengers in the cumulus cells [17]. This, in turn, influences the growth and maturation of oocytes as well as follicle selection in the ovaries [8,9,18,19,20,21,22]. Subsequently, serotonin is involved in the regulation of cell cycle, proliferation, and apoptosis in the early mouse embryo during preimplantation development [18,23,24].

The objective of this study was to examine the composition of the serotoninergic system in mouse embryonic cells at the preimplantation stage of development. The main focus was on the analysis of the expression of the pivotal components involved in the signaling function of serotonin, including its receptors and transporters. This was complemented by the visualization of these components and their functions. The presence and spatial distribution of serotonin and certain components of the serotonergic system, including the membrane transporter SERT and the 5-HT_1D_-receptor, have already been described for oocytes and early mouse embryos [20,22]. However, a comprehensive picture that could describe the functional role of the serotonergic system at key stages of early development remains to be established.

A notable deficiency in the current understanding of serotonin signaling is the lack of data on the vesicular transport of this neurotransmitter. This is a crucial element in deciphering its intercellular signaling function, as serotonin transport into intracellular vesicles capable of exocytosis is mediated by the vesicular monoamine transporter VMAT [25,26,27]. To evaluate the role of serotonin as an intercellular signaling molecule in early development, it is essential to assess the functional activity of VMAT using pharmacological inhibitors. Reserpine, an indole alkaloid, blocks the VMAT transporter and prevents the storage of serotonin in intracellular vesicles [28]. As a result, the accumulation of monoamines in cells is suppressed [25,29,30], and monoamine oxidases accelerate their degradation [31,32,33].

A component analysis of the serotoninergic system will facilitate the formulation of hypotheses regarding its mechanisms of functioning. Thus, one of the features of embryonic transmitter systems that requires verification is the possibility of simultaneous activation of multiple specific serotonin receptors within a single cell. The results obtained will enable a comparison of the structure of the embryonic serotoninergic system with that of the serotonin-producing cells of the nervous system and peripheral tissues of the adult organism.

## 2. Results

### 2.1. Transcriptomic Analysis of the Composition of the Serotonin Signaling System in Early Mouse Embryogenesis

**Figure 1:**
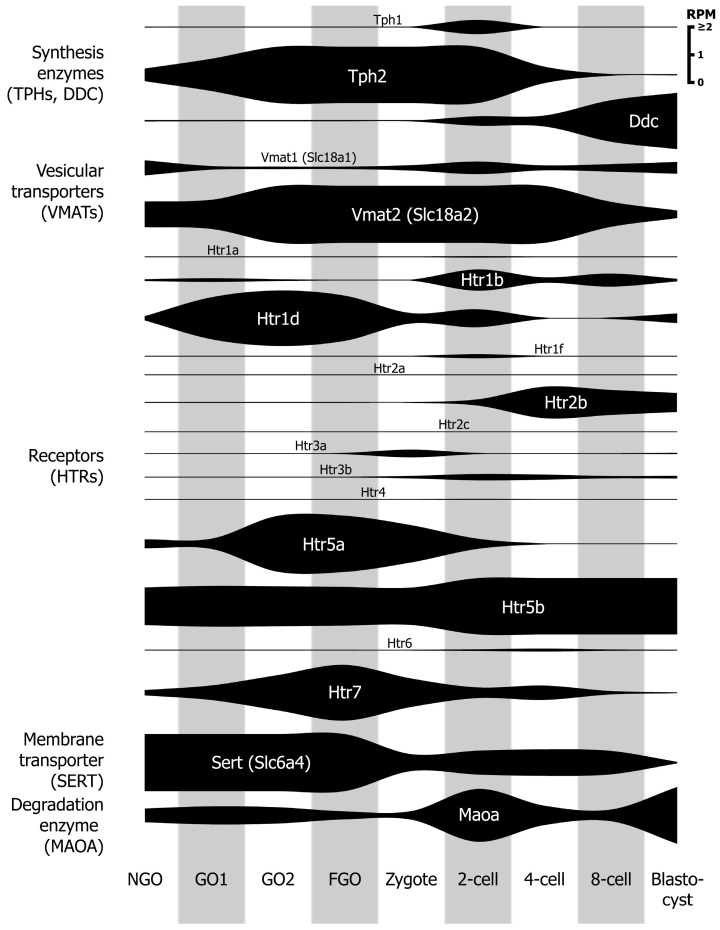
Analysis of data from transcriptomic studies [34,35] performed on mouse oocytes and preimplantation embryos. The expression level of all genes is shown on a single scale. The values are presented in units of RPM (reeds per million), representing a normalization of the data to the total amount of RNA. The shading was introduced to make it easier to distinguish between the stages of development. Stages of oogenesis according to Veselovska et al. [34]: NGO—non-growing oocytes; GO1—growing oocytes of mice 8–14 days postpartum (dpp); GO2—growing oocytes of mice 15 dpp; FGO—fully-grown oocytes of adult mice. Stages of preimplantation development according to Qiao et al. [35]: Zygote—24–26 h post hCG injection; 2-cell—46–48 h post hCG; 4-cell—54–58 h post hCG; 8-cell—68–70 h post hCG; blastocyst—94–96 h post hCG.

To determine which components of the serotonin system are active during the early stages of development, we first performed an analysis of the available transcriptomic data on the dynamics of mRNA expression at different stages of mouse oogenesis and preimplantation development [34,35]. The summarized transcriptome data are shown schematically in Figure 1 (and Table S1), which reflects the number of gene transcripts of serotonin receptors and transporters at different developmental stages.

The available data showed that the expression of enzymes involved in serotonin synthesis exhibited a sequential pattern. Throughout oogenesis and up to the 4-cell stage, high mRNA expression of tryptophan hydroxylase *Tph2*, which is responsible for the first step of the biochemical synthesis chain, was observed. At the same time, the second enzyme, aromatic amino acid decarboxylase *Ddc*, did not reach a safe expression level until the 8-cell stage of the embryo. In addition, during oogenesis and early development, the mRNA of the membrane-bound serotonin transporter *Sert* (*Slc6a4*) and the vesicular monoamine transporter *Vmat2* (*Slc18a2*), as well as the most important enzyme of serotonin degradation, monoamine oxidase A *Maoa*, were expressed at high levels.

Of the 14 serotonin receptors identified in the mouse, several were highly expressed during oogenesis and in the early stages of development. For example, the mRNA of the receptor *Htr5b* was highly expressed throughout oogenesis and up to the blastocyst stage, while *Htr1d*, *Htr5a,* and *Htr7* were maximally expressed during oogenesis and *Htr1b* and *Htr2b* during the cleavage period.

From this, it can be concluded that the crucial components that ensure the function of serotonin as a signaling molecule are expressed from the earliest stages of development, namely the oocyte and the single-cell mouse embryo. The present study focused primarily on analyzing the expression and functional activity of the vesicular monoamine transporter VMAT2 and several membrane-bound serotonin receptors, namely 5-HT_1B_, 5-HT_1D_, 5-HT_2B_, and 5-HT_7_.

### 2.2. Visualization of the Components of the Serotonin Signaling System in Early Mouse Embryogenesis

**Figure 2:**
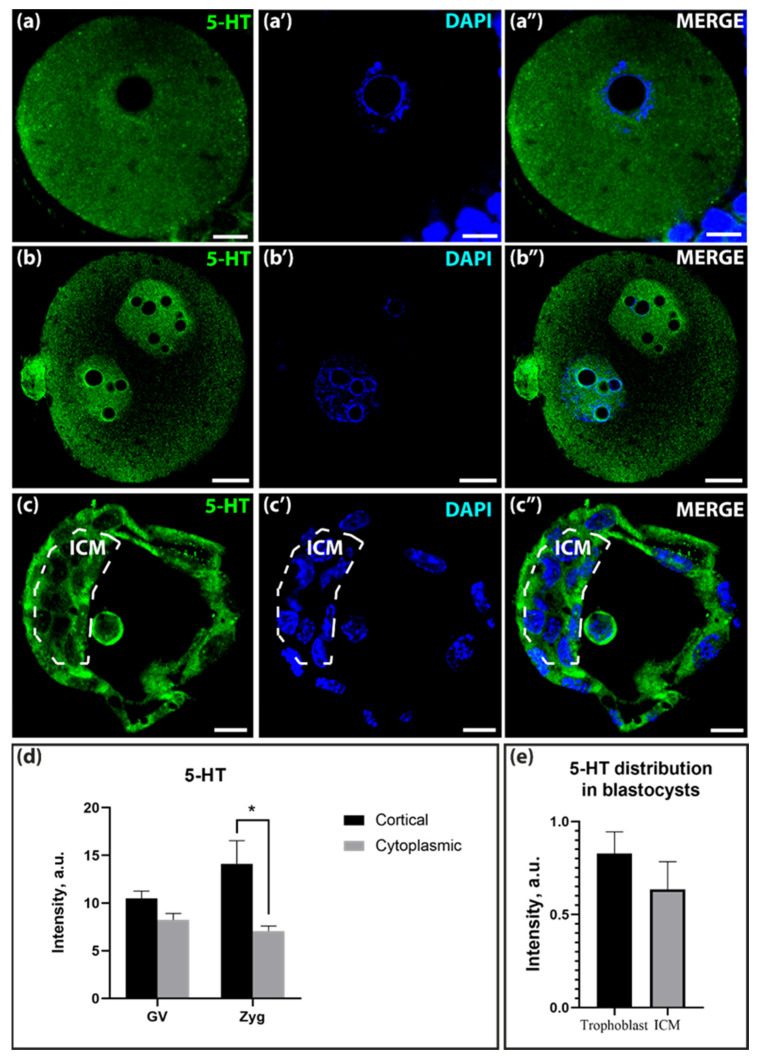
Distribution of serotonin in mice GV-oocytes, zygotes, and blastocysts. (**a**–**a″**)—distribution in GV-oocytes; (**b**–**b″**)—distribution in zygotes; (**c**–**c″**)—distribution in blastocysts. The dotted line marks the area of the inner cellular mass (ICM). Scale bar–10 μm. (**d**)—Ratio of serotonin in the cortical and cytoplasmic layers in GV-oocytes and zygotes in signal intensity arbitrary units (a.u.). Asterisks indicate significant differences between the cortical and cytoplasmic cell layers using two-way ANOVA, * *p* < 0.05; (**e**)—ratio of serotonin in ICM and trophoblast in blastocysts in a.u.

Immunohistochemistry was chosen to visualize the components of the serotonergic system in oocytes and embryos because it is a method with a high degree of specificity and reproducibility. This approach allows a quantitative assessment of the content of the antigens studied, taking into account their spatial localization. The distribution of serotonin, visualized with specific antibodies, in germinal vesicle (GV) oocytes and early mouse embryos was similar: the transmitter was detected everywhere in the cells in the form of a small-point immunoreaction (Figure 2a–a″,b–b″,c–c″). Quantitative analysis showed that the 5-HT content was slightly higher in the cortical layer of the GV-oocytes than in the subcortical cytoplasm. In zygotes, this difference was even more pronounced and statistically significant (Figure 2d). At the blastocyst stage, the 5-HT content was slightly higher in the trophoblast cells than in the inner cell mass (ICM) (Figure 2e). In some cases, serotonin was observed within the nuclei, where the nucleoplasm was stained, and there was no co-localization with chromatin (Figure 2b).

**Figure 3:**
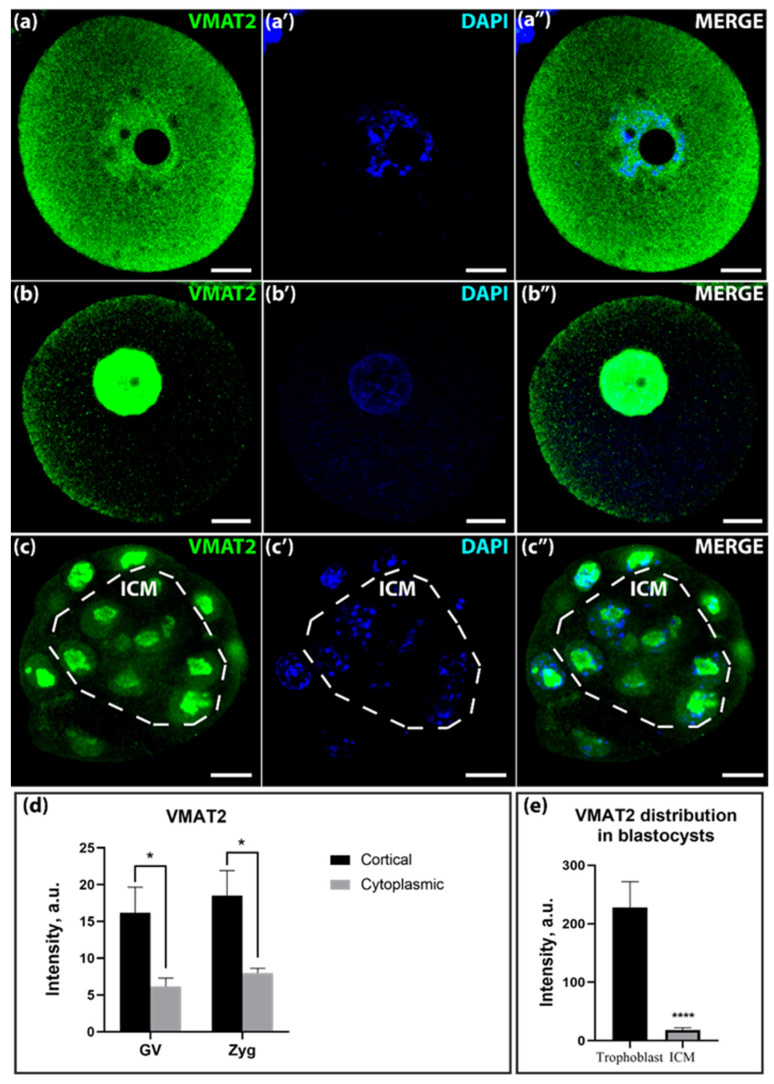
Distribution of VMAT2 in GV-oocytes, zygotes, and blastocysts of mice. (**a**–**a″**)—distribution in GV-oocytes; (**b**–**b″**)—distribution in zygotes; (**c**–**c″**)—distribution in blastocysts. The dotted line marks the area of the ICM. Scale bar–10 μm. (**d**)—Ratio of VMAT2 in the cortical and cytoplasmic layers in GV-oocytes and zygotes in a.u. Asterisks show significant differences between cortical and cytoplasmic layers using two-way ANOVA, * *p* < 0.05; (**e**)—Ratio of VMAT2 in ICM and trophoblast in blastocysts in a.u. Asterisks show significant differences between cortical and cytoplasmic cell layers using the Wilcoxon test, **** *p* < 0.0001.

The vesicular monoamine transporter VMAT2 was much more clearly visible in the cortical layer in the GV-oocyte and zygote stages (Figure 3a–a″,b–b″,d). At the blastocyst stage, VMAT2 was concentrated to a greater extent in trophectoderm cells and at very low levels in the ICM (Figure 3c–c″,e). Remarkably, the anti-VMAT2 antibodies showed strong staining of the nucleoplasm in addition to the cortical cell layer.

**Figure 4:**
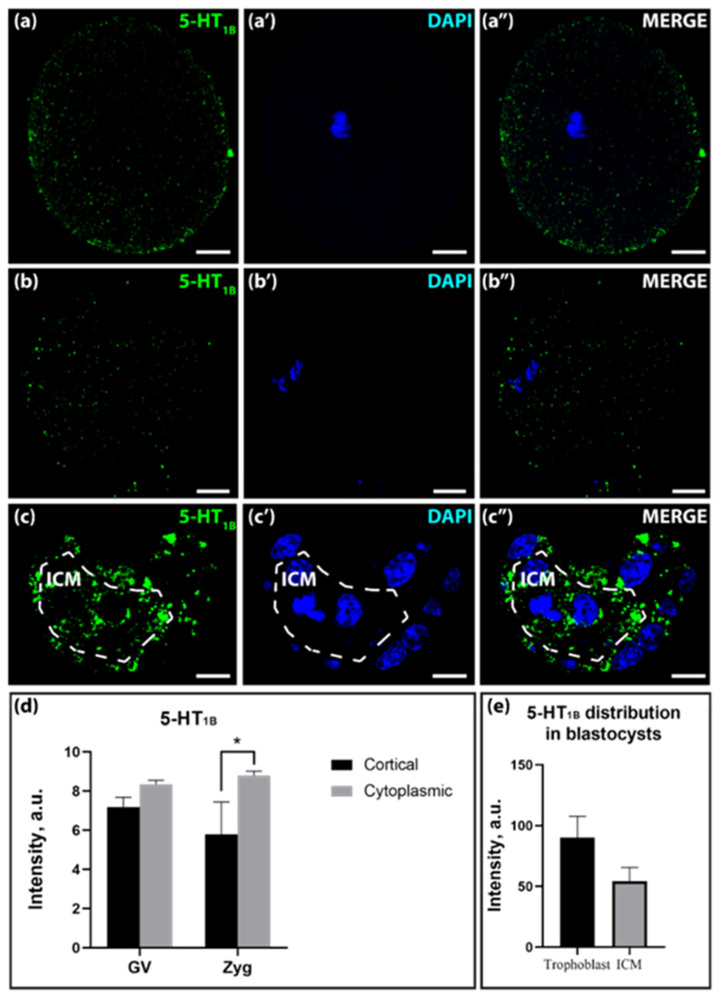
Distribution of the 5-HT_1B_-receptor in GV-oocytes, zygotes, and blastocysts of mice. (**a**–**a″**)—distribution in GV-oocytes; (**b**–**b″**)—distribution in zygotes; (**c**–**c″**)—distribution in blastocysts. The dotted line marks the area of ICM. Scale bar–10 μm. (**d**)—Ratio of 5-HT_1B_-receptor immunoreactivity in the cortical and cytoplasmic layers in GV-oocytes and zygotes in a.u. Asterisks indicate significant differences between cortical and cytoplasmic cell layers using two-way ANOVA, * *p* < 0.05; (**e**)—Ratio of 5-HT_1B_ in ICM and trophoblast in blastocysts in a.u.

According to the data on 5-HT_1B_ receptor mRNA expression, the immunoreactivity of this protein was quite weak in the GV-oocyte and zygote stages. During development, the pattern of 5-HT_1B_-receptor immunoreactivity changed. At the GV-oocyte stage, the immunoreactivity of this receptor showed an even distribution throughout the cell volume, with the exception of a slightly stronger presence in the cytoplasmic layer compared to the cortical layer (Figure 4a–a″,d). In the zygote stage, this difference was pronounced and statistically significant (Figure 4b–b″,d). In the blastocyst stage, immunoreactivity was slightly more intense in the trophoectoderm than in the ICM, but this difference was not statistically significant (Figure 4c–c″,e).

**Figure 5:**
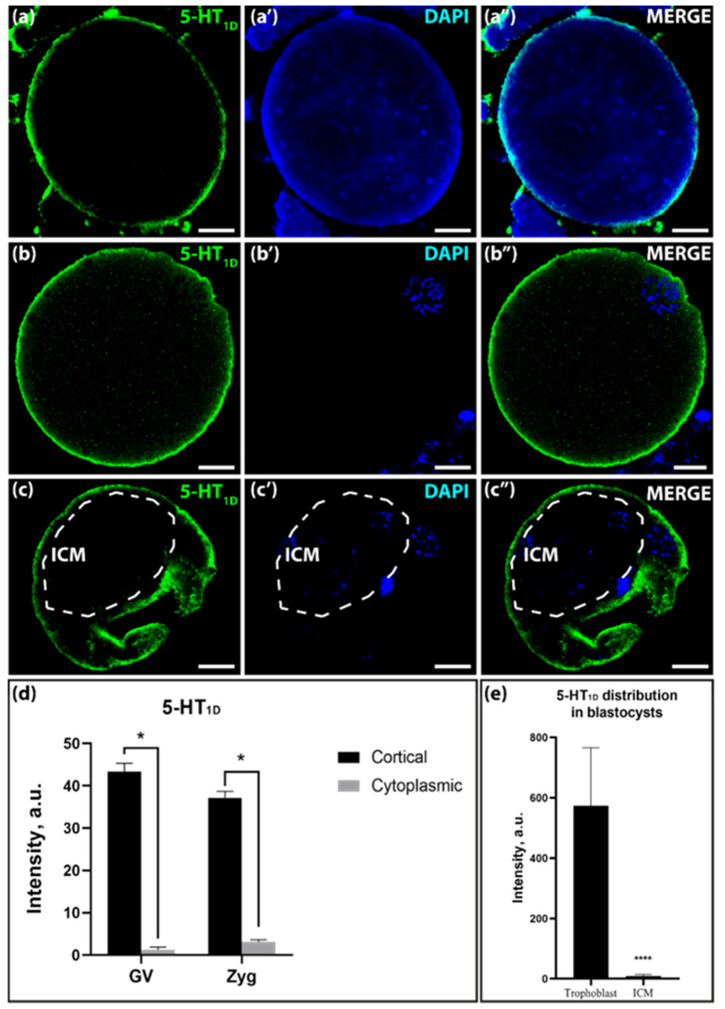
Distribution of 5-HT_1D_ in mice GV-oocytes, zygotes, and blastocysts. (**a**–**a″**)—distribution in GV-oocytes; (**b**–**b″**)—distribution in zygotes; (**c**–**c″**)—distribution in blastocysts. The dotted line marks the area of the ICM. Scale bar–10 μm. (**d**)—ratio of 5-HT_1D_ in the cortical and cytoplasmic layers in GV-oocytes and zygotes in a.u. Asterisks indicate significant differences between cortical and cytoplasmic cell layers using two-way ANOVA, * *p* < 0.05; (**e**)—ratio of 5-HT_1D_ in ICM and trophoblast in blastocysts in a.u. Asterisks indicate significant differences between cortical and cytoplasmic cell layers using the Wilcoxon test, **** *p* < 0.0001.

The spatial distribution of the 5-HT_1D_-receptor differed significantly from other markers. The immunoreactive particles in the stages of the GV-oocyte and zygote were very unevenly visible in the cell, concentrated in the narrow cortical layer of the cell, most likely covering the plasma membrane itself and almost absent in the depths of the cytoplasm (Figure 5a–a″,b–b″,d).

This initial pattern was maintained in the blastocyst stage: immunoreactivity was detected in the apical part of the trophectoderm cells, whereas we observed virtually no signal in the ICM cells (Figure 5c–c″,e). All these differences were statistically significant.

**Figure 6:**
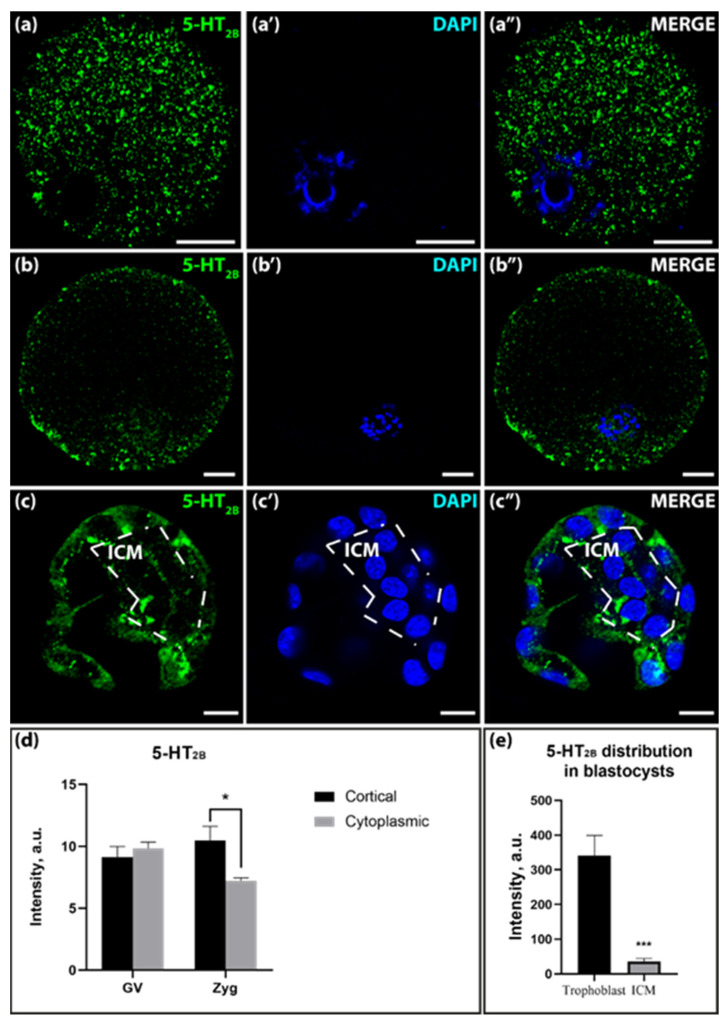
Distribution of 5-HT_2B_ in GV-oocytes, zygotes, and blastocysts of mice. (**a**–**a″**)—distribution in GV-oocytes; (**b**–**b″**)—distribution in zygotes; (**c**–**c″**)—distribution in blastocysts. The dotted line marks the area of ICM. Scale bar–10 μm. (**d**)—Ratio of 5-HT_2B_ in the cortical and cytoplasmic layers in GV-oocytes and zygotes in a.u. Asterisks denote significant differences between cortical and cytoplasmic cell layers using two-way ANOVA, * *p* < 0.05; (**e**)—ratio of 5-HT_2B_ in ICM and trophoblast in blastocysts in a.u. Asterisks denote significant differences between cortical and cytoplasmic cell layers using the Wilcoxon test, *** *p* < 0.0001.

The distribution of the 5-HT_2B_-receptor, whose mRNA expression is very low or absent in the oocyte and zygote, showed a similar pattern to the distribution of the 5-HT_1B_-receptor at these stages, but the signal intensity was visually much higher for the 5-HT_2B_-receptor. The immune response in the oocyte was detected in the form of coarse granular structures that were evenly distributed in the cytoplasm of the cell (Figure 6a–a″,d). In the zygote stage, the immunoreactivity was concentrated in the cortical compartment, and the signal intensity was significantly higher than in the cytoplasm (Figure 6b–b″,d). In the blastocyst stage, a clear signal was observed in both the trophoblast cells and the ICM cells, with receptor immunoreactivity being significantly higher in the trophoblast cells (Figure 6c–c″,e).

**Figure 7:**
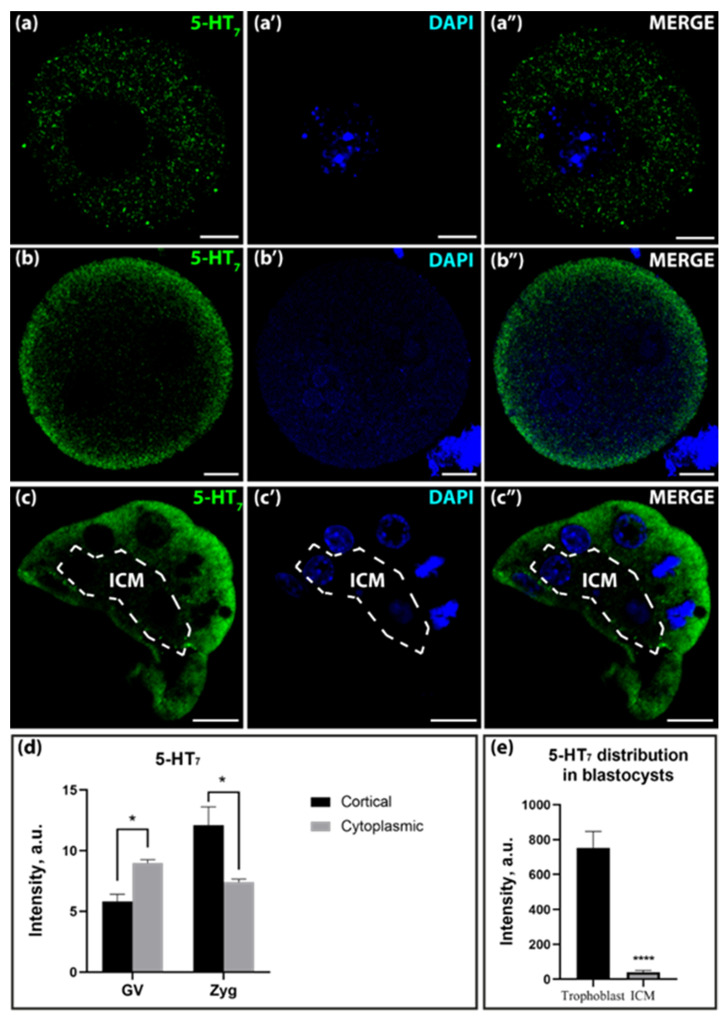
Distribution of 5-HT_7_ in GV-oocytes, zygotes, and blastocysts of mice. (**a**–**a″**)—distribution in GV-oocytes; (**b**–**b″**)—distribution in zygotes; (**c**–**c″**)—distribution in blastocysts. The dotted line marks the area of ICM. Scale bar–10 μm. (**d**)—Ratio of 5-HT_7_ in the cortical and cytoplasmic layers in GV-oocytes and zygotes in a.u. Asterisks indicate significant differences between cortical and cytoplasmic cell layers using two-way ANOVA, * *p* < 0.05; (**e**)—ratio of 5-HT_7_ in ICM and trophoblast in blastocysts in a.u. Asterisks show significant differences between cortical and cytoplasmic cell layers using the Wilcoxon test, **** *p* < 0.0001.

The 5-HT_7_ receptor was visible in the entire cell volume in the GV-oocyte stage, whereby the intensity in the cytoplasmic layer was significantly higher than in the cortical layer (Figure 7a,a′,d). In the zygote stage, the immunoreactivity against the 5-HT_7_-receptor showed a pronounced shift toward the cortical part of the cell (Figure 7b,b′,d). In both stages, the differences in signal intensity in the cortical and cytoplasmic layers are significant. In the blastocyst stage, the intensity of the immunoreactive signal decreases and is mainly visible in trophoblast cells (Figure 7c,c′,e).

### 2.3. Functional Analysis of Vesicular Transport of Serotonin During Mouse Oogenesis

As shown above, the expression of mRNA and protein of the vesicular monoamine transporter VMAT2 was at a high level during oogenesis and early development. To assess the functional activity of the vesicular transporter mechanism in maturing oocytes, experiments were performed with the specific blocker reserpine, and then the serotonin content in the ovary and maturing oocytes was analyzed.

**Figure 8:**
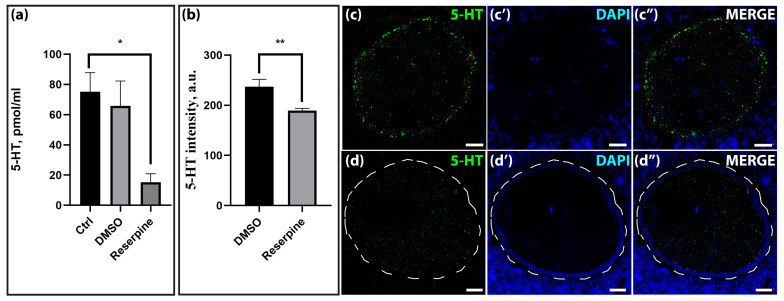
Effect of a 7-day administration of reserpine (1 mg/kg) on the serotonin content in ovaries. (**a**)—HPLC analysis of serotonin content in ovaries of control (Ctrl and DMSO) and experimental (reserpine) females. Asterisks indicate significant differences between groups (Kruskal–Wallis test), * *p* < 0.05. (**b**)—Quantification of anti-serotonin immunoreactivity in oocytes from control (DMSO) and experimental (reserpine) ovarian tissue cryosections. Asterisks indicate significant differences between the groups (Mann–Whitney test, ** *p* < 0.005). (**c**–**c″**)—immunohistochemical detection of serotonin in ovarian tissue cryosections of the control group (DMSO). (**d**–**d″**)—immunohistochemical detection of serotonin in cryosections of ovarian tissue from the experimental group (reserpine). The dotted line marks the oocyte. Scale bar 10 µm.

Quantitative analysis using the HPLC method revealed a significant reduction in serotonin content in the ovaries of reserpine-treated females, with levels reaching only 20% of the control values (Figure 8a). Immunostaining of serotonin in ovarian tissue showed a significant reduction in the serotonin levels in the oocytes of growing follicles (Figure 8b,c–c″,d–d″).

**Figure 9:**
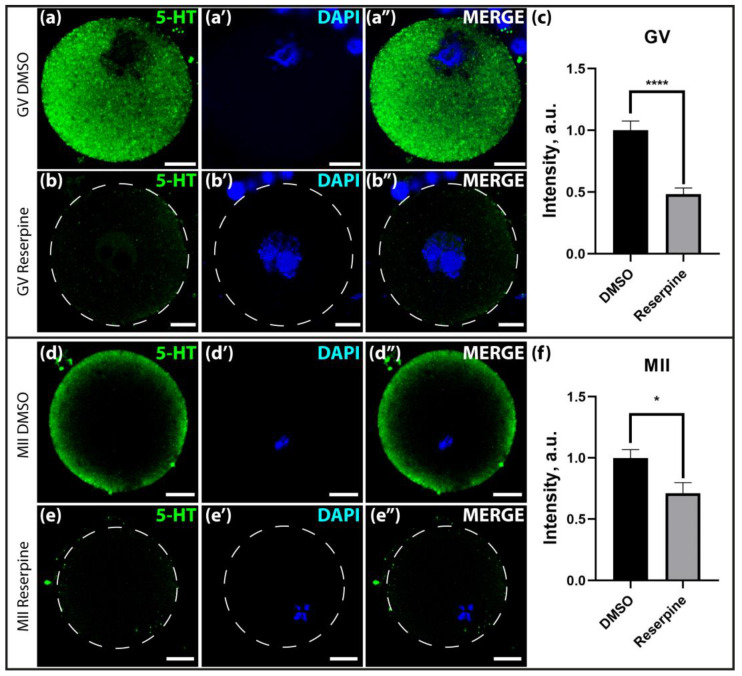
Effect of a 7-day administration of reserpine (1 mg/kg) on the serotonin content in oocytes. (**a**,**b**)—immunohistochemical detection of serotonin in GV-oocytes of the control DMSO group (**a**–**a″**) and the experimental reserpine group (**b**–**b″**). (**c**)—Quantification of anti-serotonin immunoreactivity in GV-oocytes of the control (DMSO) and experimental (reserpine) groups. (**d**,**e**)—immunohistochemical detection of serotonin in MII-oocytes of the control DMSO group (**d**–**d″**) and the experimental reserpine group (**e**–**e″**). (**f**)—Quantification of anti-serotonin immunoreactivity in the MII-oocytes of the control (DMSO) and experimental (reserpine) groups. The dotted line marks the oocyte. Scale bar 10 µm. The data have been normalized in relation to the DMSO group, with a mean value of 1. The asterisks indicate significant differences between the groups (Mann–Whitney test, * *p* < 0.05, **** *p* < 0.0001).

A significant decrease in serotonin immunoreactivity was observed in immature GV-oocytes (Figure 9a–c) isolated from the ovaries and in mature MII- oocytes (Figure 9c–f) isolated from the oviducts of mice treated with reserpine for one week. Compared to the control group that had received DMSO injections, the oocytes showed a significant decrease in serotonin content after reserpine treatment, by almost 50% in GV-oocytes and 30% in MII-oocytes. It is important to note that in most cases, not only the overall serotonin content decreased, but also its pronounced localization in the cortical region of the oocytes was virtually eliminated. This indicated the existence of a vesicular compartment within the oocyte where the accumulation of serotonin takes place as a consequence of VMAT activity. This result confirmed the functional activity of the vesicular transport of monoamines in the stages studied.

## 3. Discussion

The first aim of this study was to investigate the presence and dynamics of expression of the key components of the mouse embryonic serotoninergic system. Analysis of the transcriptomic data reveals that the mRNAs of several serotonin receptors, including 5-HT_1D_, 5-HT_5A_, 5-HT_5B_, and 5-HT_7_, are expressed at remarkable levels in the oocyte, zygote, and subsequent stages of cleavage divisions. These transcripts are obviously of maternal origin, as this is the only possibility for their presence prior to the maternal-to-zygotic transition. In addition, significant expression of 5-HTR_1B_ is observed at the 2-cell stage and of 5-HT_2B_ at the 4-cell stage, which is probably already associated with activation of the zygotic genome.

The immunohistochemical analysis, conducted using specific antibodies against 5-HT_1D_, 5-HT_1B_, 5-HT_2B,_ and 5-HT_7_ receptors, has revealed the presence of these proteins during the oogenesis and embryogenesis of the mouse. The high level of immunoreactivity, differences, and changes in their localization during development may indicate that these receptors are functionally active. Based on the combination of transcriptomic data and the results of immunocytochemical imaging, we can conclude with certainty that serotonin receptors identical to those that are functionally active in adult cells are present in the embryonic stages.

The distribution of immunoreactivity for the 5-HT_1B_ and 5-HT_2B_ receptors in the GV-oocyte and zygote stages is cytoplasmic, whereas a cortical pattern is more pronounced in the zygote stage. At the blastocyst stage, the signaling of 5-HT_1B_ receptors is significantly increased in the trophoblast cells compared to the ICM. A similar pattern was observed with respect to the 5-HT_7_ receptor. The activity of the receptors located in the cytoplasm is still controversial in the scientific community. One possible explanation is that they are synthesized and stored receptor proteins that are not functionally active. An alternative hypothesis derives from the evolutionary perspective that transmitters are primarily intracellular regulators of synthetic processes [36], which could still have these functions today. There is increasing evidence that G protein-coupled receptors (GPCRs) localized in the membranes of the endosomes, nucleus, Golgi apparatus, endoplasmic reticulum, mitochondria, and cell division compartments are also able to signal from intracellular compartments [37]. The intracellular activity of the L-DOPA receptor GPR143 has been demonstrated with high confidence in cells of the retinal pigment epithelium [38]. Furthermore, this assumption is supported by a large body of evidence for the action of transmitters and their antagonists, which must necessarily enter embryonic cells. In sea urchin blastulae, dopamine and the dopamine receptor D1 are highly co-localized in granules of 1–2 μm in diameter that are associated with the basal bodies of cilia [39]. In the context of our work, it is important to note that a crucial factor influencing the intracellular activity of membrane receptors is their compartmentalization [40].

The 5-HT_1D_-receptor, in contrast to all others, is clearly localized in the cortical layer during oo- and embryogenesis, and the differences in signal intensity compared to subcortical cytoplasm are significant in all cases. In early mouse embryos, the localization of serotonin, diffused in GV-oocytes, clearly shifts to the cortical layer. This finding is consistent with the available data on the mRNA expression of this receptor in preimplantation mouse embryos and the negative effects of its antagonists on early development [41]. The presence of serotonin receptors in the plasma membrane and transporter in the submembrane layer of mouse embryos also supports the suggestion that they are involved in the interactions between blastomeres, similar to those in sea urchin embryos [42]. The cortical cytoskeleton may represent a crucial target of such regulatory processes, as evidenced by observations in both nerve [43] and embryonic cells [44].

It is also known that the simultaneous expression of several functional receptors for one transmitter is observed in early embryogenesis [22,34,35]. However, we are unaware of any data on the simultaneous presence of multiple types of functional receptors for the same transmitter within a single-cell embryo, although we mentioned this possibility back in 2012 [24]. A synthesis of the data on differential localization and expression dynamics suggests that the various receptor types may perform specific functions in different intracellular and intercellular signaling pathways. These functions may include intracellular metabolic processes, the oocyte–cumulus interaction, interblastomere interaction, and other processes. Prior research has also established a correlation between serotonin and the activation of the zygotic genome in mammals, a phenomenon linked to epigenetic histone modifications [9,45]. To substantiate these hypotheses, future studies should undertake direct functional assessments of receptor activity in specific biological processes.

The second main objective of this study is to evaluate the functional activity of vesicular transport in early mammalian development. Transcriptome analysis reveals that the mRNA for the vesicular transporter *Vmat2* is expressed at elevated levels during oogenesis and embryogenesis. Furthermore, immunohistochemical evidence reveals that the VMAT2 transporter is present at the GV-oocyte, zygote, and blastocyst stages, with the highest intensity observed specifically at the GV-oocyte stage (Figure 3a). It is noteworthy that the spatial distribution of serotonin and VMAT2 transporter immunoreactivity shows a comparable pattern in oocytes and zygotes. This finding, in conjunction with the increased mRNA expression of this transporter, suggests that VMAT2 may serve as a primary intracellular serotonin transporter, especially during the early stages of embryonic development [22].

In our experiments, we attempted to evaluate the effect of reserpine on serotonin levels prior to fertilization. The use of reserpine in the in vivo model introduces potential limitations to the interpretation of the results due to the possibility of adverse effects. However, the continuity of the estrous cycle in females and the production of ovulated MII-oocytes indicate that reserpine does not exert any critical effects on the regulation of oogenesis. The marked decrease in ovarian serotonin levels attributable to reserpine administration can be accurately quantified by both direct biochemical assessments using HPLC and immunohistochemical staining. This demonstrates the efficacy of the methodological approaches used. After a 7-day exposure of GV-oocytes to reserpine, analysis of the images obtained shows that the serotonin levels in these oocytes decrease almost twofold and to a slightly lesser extent in the MII-oocytes. This difference, in effect, can probably be explained by the fact that the serotonin levels are much higher in the oviduct than in the ovary [46]. It can, therefore, be assumed that there is a more active uptake of serotonin by the work of the SERT transporter in the oviduct compared to the ovary, as serotonin is necessary for the later stages of oocyte maturation and further embryonic development [19]. The immunofluorescence of both VMAT2 and serotonin decreased towards the blastocyst stage. With regard to serotonin, this could be because this transmitter, which accumulates during oogenesis in the early stages, mainly through uptake via the membrane transporter SERT (see above), is actively metabolized by the enzyme MAOA in later stages. The production of its own serotonin by the embryo begins de novo after the formation of the extraembryonic organs and the placenta [47].

It is known that serotonin can influence the maturation and selection of oocytes [19,48,49,50,51]. However, it is still unclear what biological significance the accumulation of serotonin in oocytes in the ovary has. One hypothesis is that serotonin is involved in the serotonylation of proteins [52,53,54] that accumulate during oocyte growth, which is necessary for embryo functioning before the zygotic genome is activated. Since the level of serotonin accumulation depends on the SERT and VMAT2 transporters, this process can be used to identify the oocytes that are most promising for further development. Blocking intracellular transporters, as well as a transporter in the plasma membrane, can lead to disturbances in oocyte maturation and further embryonic development. However, the full spectrum of possible effects on reproductive function and early embryonic development still needs to be thoroughly investigated. To answer these questions, further pharmacological studies need to be carried out, in particular, to block the work of various receptors and other regulatory components that influence the work of serotonin transporters.

This work clarifies and expands our understanding of the serotonin system in mammalian oogenesis and embryogenesis based on transcriptomic data on the expression of the corresponding mRNAs and immunocytochemical visualizations of its components. The findings of this study suggest that serotonin signaling may be an important factor in the early stages of embryonic development and oocyte maturation, highlighting possible mechanisms by which neurotransmitter activity may influence developmental processes. Future research should focus on elucidating the specific pathways by which serotonin influences cellular interactions and differentiation during development, as well as investigating the effects of exposure to neuroactive drugs on these crucial early stages.

## 4. Materials and Methods

### 4.1. Experimental Animals and Chemicals

Mature female ICR mice from the laboratory animal center of the Koltzov Institute of Developmental Biology of the RAS were used for the animal experiments. The animals were kept under controlled conditions (22–24 °C and 14L:10D photoperiod). The mice were given ad libitum access to food and water. The chemical used in this study was reserpine (Sigma-Aldrich, St. Louis, MO, USA). In conducting this study, we placed great emphasis on the ethical treatment of the laboratory mice, with the goal of minimizing their stress throughout the experimental procedure. In accordance with the established guidelines for animal welfare, we used gentle handling techniques aimed at reducing anxiety and fear in the animals. In addition, it is important to emphasize that all handling procedures and experimental manipulations were performed in the same way in both the control and experimental groups.

The experiments were performed in accordance with the Council Directive of the European Communities of November 1986 (86/609/EEC). All animal experiment protocols were approved by the Bioethics Committee of the N.K. Koltzov Institute of Developmental Biology of the RAS.

### 4.2. Superovulation and Oocytes and Embryo Retrieval

Adult mice aged 2 months were used for the hormone stimulation protocol. The subcutaneous injection of pregnant mare serum gonadotropin (5 IU/mouse) was administered between 3 and 4 pm. After 46–48 h, an injection of human chorionic gonadotropin (hCG, 5 IU/mouse) was performed. The ovaries and oviducts were separated 20 h after the hCG injection and washed from the blood with Dulbecco’s phosphate-buffered saline (dPBS), and cooled to 4 °C. The ovaries and oviducts were placed in fresh dPBS medium (Thermo Fisher Scientific Inc., Waltham, MA, USA). Then, the GV-oocytes were isolated by rupturing the antral follicles in the ovaries with a 29 G needle, and the zygotes were isolated by cutting open the ampullary part of the oviducts.

The resulting oocytes and zygotes, which were enclosed in oocyte–cumulus complexes, were collected with a stripping pipette and transferred to a four-well plate (Thermo Fisher Scientific Inc., Waltham, MA, USA) containing dPBS medium. Hyaluronidase solution (80 IU/mL) was then added to the zygotes, which were incubated at 37 °C for one minute to remove the cumulus cells. They were then washed three times in dPBS.

In order to obtain zygotes and blastocysts, the females were placed together with the males overnight. If a vaginal plug was detected in the morning, the development time was taken as days post coitum (dpc) 0.5. Embryos at different stages of preimplantation development were obtained at time dpc 0.5 (zygote) and dpc 3.5 (blastocyst).

### 4.3. Blockade of Vesicular Serotonin Transport

To determine the level of serotonin accumulation when the vesicular monoamine transporter VMAT2 was blocked, one of the groups of animals was administered reserpine at a concentration of 1 mg/kg body weight daily for 7 days. The present dosage has been demonstrated to be efficacious in studies examining the behavioral effects of reserpine [55]. Furthermore, the duration of exposure is sufficient to produce oocytes that have undergone maturation within the cycle when exposed to the pharmacological agent. The second group was injected with dimethyl sulfoxide (DMSO) at the same volume, as reserpine is insoluble in saline.

At the end of the experiment, one ovary was removed from each group and used for subsequent analysis by HPLC, while the second ovary was fixed to obtain cryosections for further analysis by immunohistochemistry. To determine the effect of reserpine on oocyte maturation, GV-oocytes were harvested from the ovaries, and MII-oocytes were harvested from the oviducts and fixed for further immunohistochemical analyses.

### 4.4. High-Performance Liquid Chromatography (HPLC)

The serotonin content in the ovaries was quantified using high-performance liquid chromatography (HPLC) with electrochemical detection, a highly sensitive and cost-effective method for the determination of monoamine and their metabolite concentrations, with values approaching 10⁻^9^ mol/L [56]. Frozen samples were homogenized in 120 µL of 0.1 N perchloric acid containing 10 ppm/mL of the internal standard 3,4-dihydroxybenzylamine hydrobromide. The samples were then centrifuged at 2000× *g* for 20 min at 4 °C.

HPLC separation was performed on a ReproSil-Pur, ODS-3, 4.6 × 150 mm, 3 μm pore diameter reversed-phase column (Dr. Majsch, Ammerbuch, Germany) at +30 °C and a mobile phase flow rate of 1 mL/min, maintained by a 1260 Infinity II liquid chromatograph (Agilent, Santa Clara, CA, USA). The mobile phase consisted of 0.1 M citrate-phosphate buffer, 0.3 mM sodium octanesulfonate, 0.1 mM EDTA, and 8% acetonitrile (all reagents from Sigma-Aldrich, USA), pH 2.9. The Decade II electrochemical detector (Antec Leyden, Alphen a/d Rijn, The Netherlands) was equipped with a glassy carbon working electrode (+0.85 V) and an Ag/AgCl reference electrode. The peaks of serotonin and internal standard were determined based on the retention time in the standard solution.

### 4.5. Immunocytochemistry

For immunofluorescence, GV- and MII-oocytes and embryos were fixed for 1 h at room temperature or overnight at 4 °C in 4% paraformaldehyde diluted in PBS. Subsequently, the oocytes and embryos were washed with PBS with 0.1% Triton-X100 (PBST) and treated with 1% sodium dodecyl sulfate (SDS) solution to remove the zona pellucida and then washed again in PBST. Additional samples were incubated in a blocking solution for 1 h consisting of a mixture of 3% bovine serum albumin (Sigma-Aldrich), 1% fetal calf serum (Sigma-Aldrich, St. Louis, MO, USA), 0.1% Triton X-100 (Sigma-Aldrich, St. Louis, MO, USA), 0.01% Tween-20 (Sigma-Aldrich, St. Louis, MO, USA) and 0.01 M PBS (pH 7.4).

Further samples were incubated overnight at 4 °C in the mixture of blocking solution and first antibodies. The first antibodies were used as follows: rabbit anti-5-HT antibody (Sigma-Aldrich, S5545, 1:1000), rabbit anti-VMAT2 antibody (Invitrogen, Waltham, MA, USA, PA5-77496, 1:1000), rabbit anti-5-HT_1B_ receptor antibody (ABclonal, Wuhan, China, A18285, 1:1000), rabbit anti-5-HT_1D_ receptor antibody (Invitrogen, PA5-95901, 1:1000), rabbit anti-5-HT_2B_ receptor antibody (ABclonal, A5670, 1:1000), and rabbit anti-5-HT_7_ receptor antibody (ABclonal, A1976, 1:1000). The samples were washed in PBST and incubated with the following secondary antibodies: FITC-conjugated goat anti-rabbit IgG antibody (Jackson ImmunoResearch, Cambridgeshire, UK, 111-095-003, 1:200) or goat anti-rabbit IgG antibody 555 (ABclonal, Wuhan, China, A5058, 1:300). The specificity of immunostaining in each experiment was evaluated by conducting a negative control staining without the addition of primary antibodies. The samples were then washed and placed on slides in Mowiol for microscopic analysis.

### 4.6. Image Analysis and Statistics

To determine the spatial distribution of the investigated components in the oocytes and cells of preimplantation embryos, full-size GV-oocytes, zygotes, and blastocysts were immunohistochemically labeled with specific antibodies. Using the obtained images, we analyzed the distribution of the immunopositive signal in the cytoplasm and cortical layers of the oocytes and zygotes and compared the intensity of staining of trophoblast and ICM cells in blastocysts.

We analyzed the samples with a Zeiss LSM 880 Airyscan confocal laser scanning microscope (Carl Zeiss AG, Jena, Germany). All samples of an experiment were analyzed with the same microscope and software characteristics as follows: objectives, pinhole aperture, laser intensity, and detector sensitivity values. The photomicrographs were analyzed with the software FIJI ImageJ 2.9.0/1.54f (an open-source project). Immunoreactivity was quantified by the fluorescence intensity using the mean grayscale instrument for the regions of interest.

In each experiment, 15 to 30 objects obtained in three independent experimental repetitions were stained. For each object, a single confocal image was obtained at the level of the median optical slice. Statistical analyses were performed using GraphPad Prism 8.0.1 (GraphPad Software, San Diego, CA, USA) using nonparametric criteria in light of the fact that the samples did not satisfy the requisite normality criteria. In the case of pairwise comparisons, the Mann–Whitney test for unrelated samples and the Wilcoxon test for related samples were employed. The Kruskall–Wallis test was utilized for multiple comparisons. In instances where two factors (oocyte type, signal localization) were considered in a single experiment, the analysis was conducted using an ordinary two-way ANOVA with Sidak’s multiple comparisons test. The criterion for significance was set at *p* < 0.05.

## Data Availability

The original contributions presented in the study are included in the article/Supplementary Materials, further inquiries can be directed to the corresponding author.

## Supplementary Materials

The following supporting information can be downloaded at: https://www.mdpi.com/article/10.3390/ijms252312954/s1.

## References

[1] Folk G.E., Jp L. (1988). Serotonin as a Neurotransmitter: A Review. Comp. Biochem. Physiol. Part C Comp. Pharmacol..

[2] Walther D.J., Peter J.-U., Bashammakh S., Hörtnagl H., Voits M., Fink H., Bader M. (2003). Synthesis of Serotonin by a Second Tryptophan Hydroxylase Isoform. Science.

[3] Buznikov G.A., Chudakova I.V., Zvezdina N.D. (1964). The Role of Neurohumours in Early Embryogenesis. I. Serotonin Content of Developing Embryos of Sea Urchin and Loach. Development.

[4] Koshtoyants K.S., Buznikov G.A., Manukhin B.N. (1961). The Possible Role of 5-Hydroxytryptamine in the Motor Activity of Embryos of Some Marine Gastropods. Comp. Biochem. Physiol..

[5] Buznikov G.A., Kost A.N., Kucherova N.F., Mndzhoyan A.L., Suvorov N.N., Berdysheva L.V. (1970). The Role of Neurohumours in Early Embryogenesis: III. Pharmacological Analysis of the Role of Neurohumours in Cleavage Divisions. Development.

[6] Nikishin D.A., Alyoshina N.M., Semenova M.L., Shmukler Y.B. (2017). Expression Dynamics of the Serotonergic System Components in Granulosa Cells of the Developing Ovarian Follicle and After Luteinization. Genes Cells.

[7] Shmukler Y.B., Nikishin D.A. (2022). Non-Neuronal Transmitter Systems in Bacteria, Non-Nervous Eukaryotes, and Invertebrate Embryos. Biomolecules.

[8] Dubé F., Amireault P. (2007). Local Serotonergic Signaling in Mammalian Follicles, Oocytes and Early Embryos. Life Sci..

[9] Romero-Reyes J., Molina-Hernández A., Díaz N.F., Camacho-Arroyo I. (2021). Role of Serotonin in Vertebrate Embryo Development. Reprod. Biol..

[10] Nikishin D.A., Malchenko L.A., Milošević I., Rakic L., Shmukler Y.B. (2020). Effects of Haloperidol and Cyproheptadine on the Cytoskeleton of the Sea Urchin Embryos. Biochem. Suppl. Ser. A Membr. Cell Biol..

[11] Stępińska U., Kuwana T., Olszańska B. (2015). Serotonin Receptors Are Selectively Expressed in the Avian Germ Cells and Early Embryos. Zygote.

[12] Liu L., Fu M., Pei S., Zhou L., Shang J. (2018). R-Fluoxetine Increases Melanin Synthesis Through a 5-HT1A/2A Receptor and P38 MAPK Signaling Pathways. Int. J. Mol. Sci..

[13] Nikishin D.A., Kremnyov S.V., Konduktorova V.V., Shmukler Y.B. (2012). Expression of Serotonergic System Components during Early Xenopus Embryogenesis. Int. J. Dev. Biol..

[14] Reisoli E., De Lucchini S., Nardi I., Ori M. (2010). Serotonin 2B Receptor Signaling Is Required for Craniofacial Morphogenesis and Jaw Joint Formation in *Xenopus*. Development.

[15] Basu B., Desai R., Balaji J., Chaerkady R., Sriram V., Maiti S., Panicker M.M. (2008). Serotonin in Pre-Implantation Mouse Embryos Is Localized to the Mitochondria and Can Modulate Mitochondrial Potential. Reproduction.

[16] Il’kova G., Rehák P., Veselá J., Čikos S., Fabian D., Czikkova S., Koppel J. (2004). Serotonin Localization and Its Functional Significance during Mouse Preimplantation Embryo Development. Zygote.

[17] Čikos S., Fabian D., Makarevich A.V., Chrenek P., Koppel J. (2011). Biogenic Monoamines in Preimplantation Development. Hum. Reprod..

[18] Amireault P., Dubé F. (2005). Intracellular cAMP and Calcium Signaling by Serotonin in Mouse Cumulus-Oocyte Complexes. Mol. Pharmacol..

[19] Alyoshina N.M., Tkachenko M.D., Nikishina Y.O., Nikishin D.A. (2023). Serotonin Transporter Activity in Mouse Oocytes Is a Positive Indicator of Follicular Growth and Oocyte Maturity. Int. J. Mol. Sci..

[20] Alyoshina N.M., Tkachenko M.D., Malchenko L.A., Shmukler Y.B., Nikishin D.A. (2022). Uptake and Metabolization of Serotonin by Granulosa Cells Form a Functional Barrier in the Mouse Ovary. Int. J. Mol. Sci..

[21] Amireault P., Dubé F. (2005). Serotonin and its antidepressant-sensitive transport in mouse cumulus-oocyte complexes and early embryos. Biol. Reprod..

[22] Frolova V.S., Ivanova A.D., Konorova M.S., Shmukler Y.B., Nikishin D.A. (2023). Spatial Organization of the Components of the Serotonergic System in the Early Mouse Development. Biochem. Mosc. Suppl. Ser. Membr. Cell Biol..

[23] Shmukler Y.B., Alyoshina N.M., Malchenko L.A., Nikishin D.A. (2022). The Serotonin System in Mammalian Oogenesis. Neurosci. Behav. Physiol..

[24] Shmukler Y.B., Nikishin D.A., Gowder S. (2012). Transmitters in Blastomere Interactions. Cell Interaction.

[25] Wang Y., Zhang P., Chao Y., Zhu Z., Yang C., Zhou Z., Li Y., Long Y., Liu Y., Li D. (2024). Transport and Inhibition Mechanism for VMAT2-Mediated Synaptic Vesicle Loading of Monoamines. Cell Res..

[26] Baronio D., Chen Y., Decker A.R., Enckell L., Fernández-López B., Semenova S., Puttonen H.A.J., Cornell R.A., Panula P. (2022). Vesicular Monoamine Transporter 2 (SLC18A2) Regulates Monoamine Turnover and Brain Development in Zebrafish. Acta Physiol..

[27] Kayabaşı Y., Güneş B., Erbaş O. (2021). Serotonin Receptors and Depression. JEB Med. Sci..

[28] Pidathala S., Liao S., Dai Y., Li X., Long C., Chang C.-L., Zhang Z., Lee C.-H. (2023). Mechanisms of Neurotransmitter Transport and Drug Inhibition in Human VMAT2. Nature.

[29] Wu D., Chen Q., Yu Z., Huang B., Zhao J., Wang Y., Su J., Zhou F., Yan R., Li N. (2024). Transport and Inhibition Mechanisms of Human VMAT2. Nature.

[30] Naudon L., Raisman-Vozari R., Edwards R.H., Leroux-Nicollet I., Peter D., Liu Y., Costentin J. (1996). Reserpine Affects Differentially the Density of the Vesicular Monoamine Transporter and Dihydrotetrabenazine Binding Sites. Eur. J. Neurosci..

[31] Kliman H.J., Quaratella S.B., Setaro A.C., Siegman E.C., Subha Z.T., Tal R., Milano K.M., Steck T.L. (2018). Pathway of Maternal Serotonin to the Human Embryo and Fetus. Endocrinology.

[32] Romero-Reyes J., Vázquez-Martínez E.R., Bahena-Alvarez D., López-Jiménez J., Molina-Hernández A., Camacho-Arroyo I., Díaz N.F. (2021). Differential Localization of Serotoninergic System Elements in Human Amniotic Epithelial Cells. Biol. Reprod..

[33] Nordquist N., Oreland L. (2010). Serotonin, Genetic Variability, Behaviour, and Psychiatric Disorders—A Review. Ups. J. Med. Sci..

[34] Veselovska L., Smallwood S.A., Saadeh H., Stewart K.R., Krueger F., Maupetit-Méhouas S., Arnaud P., Tomizawa S.-I., Andrews S., Kelsey G. (2015). Deep sequencing and de novo assembly of the mouse oocyte transcriptome define the contribution of transcription to the DNA methylation landscape. Genome Biol..

[35] Qiao Y., Ren C., Huang S., Yuan J., Liu X., Fan J., Lin J., Wu S., Chen Q., Bo X. (2020). High-Resolution Annotation of the Mouse Preimplantation Embryo Transcriptome Using Long-Read Sequencing. Nat. Commun..

[36] Buznikov G.A. (1990). Neurotransmitters in Embryogenesis.

[37] Fasciani I., Carli M., Petragnano F., Colaianni F., Aloisi G., Maggio R., Scarselli M., Rossi M. (2022). GPCRs in Intracellular Compartments: New Targets for Drug Discovery. Biomolecules.

[38] Bueschbell B., Manga P., Schiedel A.C. (2022). The Many Faces of G Protein-Coupled Receptor 143, an Atypical Intracellular Receptor. Front. Mol. Biosci..

[39] Katow H., Suyemitsu T., Ooka S., Yaguchi J., Jin-nai T., Kuwahara I., Katow T., Yaguchi S., Abe H. (2010). Development of a Dopaminergic System in Sea Urchin Embryos and Larvae. J. Exp. Biol..

[40] Klauer M.J., Willette B.K.A., Tsvetanova N.G. (2024). Functional diversification of cell signaling by GPCR localization. J. Biol. Chem..

[41] Veselá J., Rehák P., Mihalik J., Czikková S., Pokorný J., Koppel J. (2003). Expression of serotonin receptors in mouse oocytes and preimplantation embryos. Physiol. Res..

[42] Shmukler Y.B. (1993). On the Possibility of Membrane Reception of Neurotransmitter in Sea Urchin Early Embryos. Comp. Biochem. Physiol..

[43] Cornea-Hebert V., Watkins K.C., Roth B.L., Kroeze W.K., Gaudreau P., Leclerc N., Descarries L. (2002). Similar Ultrastructural Distribution of the 5-HT(2A) 5-HT Receptor and Microtubule-Associated Protein MAP1A in Cortical Dendrites of Adult Rat. Neuroscience.

[44] Grigor’ev N.G. (1988). The Cortical Layer of the Cytoplasm—A Possible Site of the Action of Prenervous Transmitters. Zh. Evol. Biokhim. Fiziol..

[45] Farrelly L.A., Thompson R.E., Zhao S., Lepack A.E., Lyu Y., Bhanu N.V., Zhang B., Loh Y.-H.E., Ramakrishnan A., Vadodaria K.C. (2019). Histone Serotonylation Is a Permissive Modification That Enhances TFIID Binding to H3K4me3. Nature.

[46] Amenta F., Vega J.A., Ricci A., Collier W.L. (1992). Localization of 5-hydroxytryptamine-like Immunoreactive Cells and Nerve Fibers in the Rat Female Reproductive System. Anat. Rec..

[47] Wu H.-H., Choi S., Levitt P. (2016). Differential Patterning of Genes Involved in Serotonin Metabolism and Transport in Extra-Embryonic Tissues of the Mouse. Placenta.

[48] Krantic S., Dube F., Quirion R., Guerrier P. (1991). Pharmacology of the Serotonin-Induced Meiosis Reinitiation in *Spisula solidissima* Oocytes. Dev. Biol..

[49] Tinikul Y., Joffre Mercier A., Soonklang N., Sobhon P. (2008). Changes in the Levels of Serotonin and Dopamine in the Central Nervous System and Ovary, and Their Possible Roles in the Ovarian Development in the Giant Freshwater Prawn, *Macrobrachium rosenbergii*. Gen. Comp. Endocrinol..

[50] Lister A., Regan C., Van Zwol J., Van Der Kraak G. (2009). Inhibition of Egg Production in Zebrafish by Fluoxetine and Municipal Effluents: A Mechanistic Evaluation. Aquat. Toxicol..

[51] Sheng Y., Wang L., Liu X.S., Montplaisir V., Tiberi M., Baltz J.M., Liu X.J. (2005). A Serotonin Receptor Antagonist Induces Oocyte Maturation in Both Frogs and Mice: Evidence That the Same G Protein-coupled Receptor Is Responsible for Maintaining Meiosis Arrest in Both Species. J. Cell. Physiol..

[52] Chan J.C., Alenina N., Cunningham A.M., Ramakrishnan A., Shen L., Bader M., Maze I. (2024). Serotonin Transporter-Dependent Histone Serotonylation in Placenta Contributes to the Neurodevelopmental Transcriptome. J. Mol. Biol..

[53] Bader M. (2019). Serotonylation: Serotonin Signaling and Epigenetics. Front. Mol. Neurosci..

[54] Voronezhskaya E.E. (2021). Maternal Serotonin: Shaping Developmental Patterns and Behavioral Strategy on Progeny in Molluscs. Front. Ecol. Evol..

[55] De Freitas C.M., Busanello A., Schaffer L.F., Peroza L.R., Krum B.N., Leal C.Q., Ceretta A.P.C., Da Rocha J.B.T., Fachinetto R. (2016). Behavioral and neurochemical effects induced by reserpine in mice. Psychopharmacology.

[56] Guiard B.P., Gotti G. (2024). The High-Precision Liquid Chromatography with Electrochemical Detection (HPLC-ECD) for Monoamines Neurotransmitters and Their Metabolites: A Review. Molecules.

